# Material Defects in Friction Stir Welding through Thermo–Mechanical Simulation: Dissimilar Materials with Tool Wear Consideration

**DOI:** 10.3390/ma16010301

**Published:** 2022-12-28

**Authors:** Debtanay Das, Swarup Bag, Sukhomay Pal, Abhay Sharma

**Affiliations:** 1 Department of Mechanical Engineering, Indian Institute of Technology Guwahati, Guwahati 781039, India; 2 Faculty of Engineering Technology, KU Leuven, Campus De Nayer, 2860 Sint-Katelijne-Waver, Belgium

**Keywords:** friction stir welding, defects in FSW, CEL approach, finite element model, dissimilar welding, solid state joining

## Abstract

Despite the remarkable capabilities of friction stir welding (FSW) in joining dissimilar materials, the numerical simulation of FSW is predominantly limited to the joining of similar materials. The material mixing and defects’ prediction in FSW of dissimilar materials through numerical simulation have not been thoroughly studied. The role of progressive tool wear is another aspect of practical importance that has not received due consideration in numerical simulation. As such, we contribute to the body of knowledge with a numerical study of FSW of dissimilar materials in the context of defect prediction and tool wear. We numerically simulated material mixing and defects (surface and subsurface tunnel, exit hole, and flash formation) using a coupled Eulerian–Lagrangian approach. The model predictions are validated with the experimental results on FSW of the candidate pair AA6061 and AZ31B. The influence of tool wear on tool dimensions is experimentally investigated for several sets of tool rotations and traverse speeds and incorporated in the numerical simulation to predict the weld defects. The developed model successfully predicted subsurface tunnel defects, surface tunnels, excessive flash formations, and exit holes with a maximum deviation of 1.2 mm. The simulation revealed the substantial impact of the plate position, on either the advancing or retreating side, on the defect formation; for instance, when AZ31B was placed on the AS, the surface tunnel reached about 50% of the workpiece thickness. The numerical model successfully captured defect formation due to the wear-induced changes in tool dimensions, e.g., the pin length decreased up to 30% after welding at higher tool rotations and traverse speeds, leading to surface tunnel defects.

## 1. Introduction

Friction stir welding (FSW) is a multiphysics welding technique that produces joints primarily through frictional heat generation and material plasticization. FSW, being a solid-state process, is tremendously effective in joining several dissimilar alloys that are difficult to join with arc welding. The dissimilar materials’ welds obtained with FSW, such as aluminum and magnesium alloys, have been applied in lightweight automobiles. The FSW of dissimilar materials (commonly known as dissimilar FSW) has been investigated with the help of experimental and numerical studies. The FSW process has been numerically modeled mainly to predict heat generation, material flow, stress formation, and strain, etc. [1]. The numerical prediction of such results can lead to an easy and cost-effective prediction of weld quality, which can further improve the application of dissimilar welds in the manufacturing industry.

Numerical-simulation-based studies of dissimilar FSW have been conducted from several perspectives, for instance, steady-state and transient analysis [2]; the use of experimentally measured thermophysical properties in simulation [3]; the prediction of grain size [4]; the effect of the change in tool position [5], the pin profile [6], and the plate position [7]; the material flow for different combinations of dissimilar alloys (e.g., aluminum and brass [8], aluminum-to-copper alloys [9], aluminum-to-magnesium alloys [10], Al–Mg alloy and low carbon steel [11], and copper and duplex stainless steel [12]; intermetallic compound formation [13]; and cooling-assisted dissimilar FSW [14], etc. 

Experimental studies to represent tool–material interaction behavior in FSW are cumbersome and often impossible. Hence, the numerical simulation of materials’ defects (mainly surface and subsurface) using a coupled Eulerian–Lagrangian approach is a solution for establishing an FSW system. The need to clearly and accurately predict thermo-mechanical responses has increased the scope of the applications of numerical models to investigate the FSW process in depth. For instance, the surface and sub-surface defects in FSW of similar materials have been numerically modeled [15]. Ajri et al. [16] predicted the formation of cavities and tunnel defects. Surface tunnels or partial joints result from improper process parameters that can lead to insufficient heat generation and material mixing [17]. Excessive flash formation is another commonly occurring defect in FSW [18], which is generally observed on the retreating side (RS) due to the deposition of material behind the tool on the RS. The defect prediction in dissimilar FSW was statistically quantified [19]; however, numerical-simulation-based prediction has not yet been studied. 

A literature review indicated the need for further investigations to specifically advance the numerical modeling of FSW:

(a) Numerical models are available to predict FSW outcomes with similar materials; however, studies on the defect prediction and material mixing in dissimilar FSW are lacking;
(b) The tool wear in the numerical simulation of defect formation has not been considered in research. The material flow and change in weld quality with modified pin profiles due to tool wear have been largely unexplored. The reported numerical models of the FSW consider the dimensions of an unaltered tool. In contrast, the tool wears while welding, which produces defective joints due to improper material mixing and inclusions from tool wear [20].

Our objective in this study was two-fold. First, we aimed to enhance the understanding of material mixing and defect prediction in dissimilar FSW through an experimental numerical investigation for a candidate pair of Al–Mg alloys. Second, we aimed to enrich the model used for predicting defects in dissimilar FSW by considering the tool wear phenomenon. Thus, our study contributes to the gap knowledge by expanding the existing FSW model to both the defect prediction in FSW of dissimilar materials and the consideration of tool wear in the generation of defects.

In this study, we developed a numerical model based on the CEL approach that is capable of predicting various surface and subsurface defects and material mixing associated with dissimilar FSW. Subsequently, we produced FSW samples for dissimilar Al–Mg alloys for different tool rotation and traverse speeds. The results obtained with these samples corroborated the numerical model results and quantified the tool modification due to wear under different operating conditions. The predicted results of material mixing were verified by the elemental mapping obtained with energy-dispersive X-ray spectroscopy (EDS). We confirmed the flash formation and tunnel defects predicted by the numerical model through the visual inspection of the weld surface and cross-section. We also used the developed model to determine the effect of changes in the position of the plate during dissimilar welding, i.e., Al and Mg alloy workpieces on the advancing side (AS) and the retreating side (RS), respectively, and vice versa. Finally, we analyzed the influence of operating conditions on tool wear. We demonstrated how a tool that has been modified due to wear can generate surface and subsurface defects. In contrast to the method currently used, i.e., which considers that the tool remains unaltered irrespective of wear, the method proposed here innovatively incorporates the effect of the tool wear on predicting defects in FSW. The advantage is the realistic estimation of defect prediction. 

The following sections present materials and methods, followed by a description of the numerical model. Subsequently, we discuss the study results in the context of material mixing, defect prediction, the effect of plate position, and the effect of tool wear for dissimilar FSW. 

## 2. Materials and Methods

**Figure 1:**
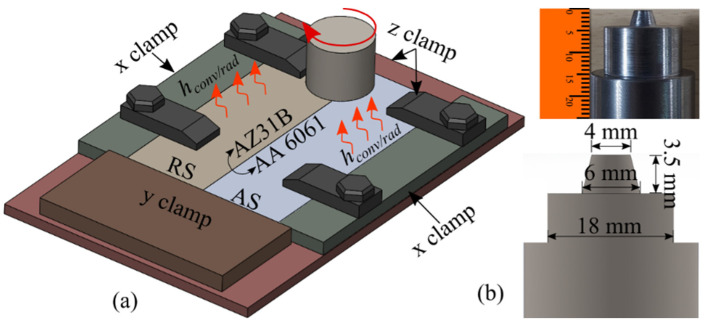
(**a**) A schematic of dissimilar FSW and (**b**) the detailed dimensions of the tool used for FSW.

We used the aluminum alloy AA6061 and magnesium alloy AZ31B for this study. Before welding, we polished the faying edges of the workpieces thoroughly using emery paper, which we then cleaned with acetone. The material compositions (wt%) of both base materials, as obtained by the EDS analysis, are provided in Table 1. We used 100 mm × 60 mm × 4 mm plates to perform dissimilar FSW. A schematic of the FSW setup is depicted in Figure 1a. For FSW, we used H-13 tool steel with a circular shoulder and conical pin. The chemical composition of the tool, as obtained by EDS analysis, is presented in Table 2.

The shoulder diameter was 18 mm, and the conical pin had major and minor diameters of 6 and 4 mm, respectively (Figure 1b). The tool plunge depth into the workpiece was 0.3 mm. We used 900 and 1200 rpm for the welding. The tool traverse speeds were 30, 60, and 90 mm/min. The FSW process parameters used in the welding are provided in Table 3. We used a stereo microscope (Nikon, model: SMZ25) to capture and analyze the tool wear after each welding. The tool had a coating of base material on the tool shoulder and the pin after every welding. We measured the dimensions of the tool before and after removing the coating layer. The precise determination of the tool shape and size, and more importantly, of the tool pin, is vital for a high-quality weld. We analyzed the material adhering to the tool and the material mixing using a field-emission scanning electron microscope (FESEM) and energy-dispersive spectroscopy (EDS) analysis, performed using a Sigma 300 (Zeiss), and investigated IMCs formation through XRD analysis performed using a Rigaku X-ray diffractometer with CuKα radiation (λ = 1.54Å). We employed a scanning rate of 0.05°/s, a scanning range of 20–90°, and used a power level of 9 kW in the study.

## 3. The Theoretical Model

We modeled the workpiece as an Eulerian body and the tool as a Lagrangian body. We created a void domain over the Eulerian workpiece domain to capture the flash formation during welding. The void domain was a third Eulerian domain without any material assigned to it at the initiation of the simulation, i.e., the elements of the void domain were initially empty. As the tool plunged into the workpiece, the material moved out to form the flash. The empty void domain captured this outgoing material from the workpiece domain. If the void domain is not modeled, the material moving out of the workpiece is lost to the ambient surroundings for further analysis. We used the volume of fluid (VOF) technique to identify the distinct materials during dissimilar FSW. In the coupled Eulerian and Lagrangian (CEL) approach, the VOF approach is used, in which the Eulerian volume fraction (EVF) of each element is tracked. The EVF ranges between 0 and 1, i.e., it is entirely void and completely filled, respectively. 

We modeled FSW following the CEL technique using commercially available FEM-based software ABAQUS 2017. The conventional thermal validation of the model is provided elsewhere [15]. The current model focused on material flow, material mixing, and the evolution of defects in dissimilar FSW. To model the dissimilar FSW, the material flowed between two Eulerian bodies placed adjacent to one another with adjoining faying edges. The prediction of the aforementioned responses and their validation is described in subsequent sections. We used the Johnson–Cook (JC) material and damage models to simultaneously predict the plastic deformation and defect formation of both materials with the traverse motion of the rotating tool, as defined by Equation (1):

(1) $$\bar{\sigma }=\left[A+B\;{\bar{\varepsilon }}^{n}\right]\left[1+C\ln\left(\frac{\dot{\bar{\varepsilon }}}{\dot{\varepsilon_{0}}}\right)\right]\left[1-{\left(\frac{T-T_{room}}{T_{melt}-T_{room}}\right)}^{m}\right]$$

where $\bar{\sigma }$ is the flow stress, $\bar{\varepsilon }$ is the plastic strain, $\dot{\bar{\varepsilon }}$ is the effective strain rate, ${\dot{\varepsilon }}_{0}$ is the reference strain rate ($1\;s^{-1}$), $T_{room}$ is room temperature, $T_{melt}$ is the melting temperature, A is the yield stress of the material, B is the strain hardening modulus, n is the work-hardening exponent, C is the strain rate hardening, and m is the thermal softening coefficient:

(2) $$D=\frac{\bar{\varepsilon }}{{\bar{\varepsilon }}_{f}}$$

(3) $${\bar{\varepsilon }}_{f}=\left[d_{1}+d_{2}\exp\left(d_{3}\frac{p}{\bar{\sigma }}\right)\right]\left[1+d_{4}\ln\left(\frac{\dot{\bar{\varepsilon }}}{\dot{\varepsilon_{0}}}\right)\right]\left[1+d_{5}\left(\frac{T-T_{room}}{T_{melt}-T_{room}}\right)\right]$$

where, D is the damage parameter, $\bar{\varepsilon }$ is the effective plastic strain, ${\bar{\varepsilon }}_{f}$ is the plastic strain in failure, $d_{1}-d_{5}$ is material dependent constants, p and $\bar{\sigma }$ are the pressure and von-mises stress. The application of the JC damage in the developed numerical model is described previously by the authors elsewhere [15]. The JC model material constants are provided in Table 4 and Table 5.

The thermo-physical properties used for the current work are presented in Table 6 and Table 7. The frictional contact between the tool and workpiece is the primary source of heat generation, whereas plastic heat generation aids in raising the temperature of the deforming material. The heat generated during FSW is given by:

(4) $$\partial Q=\omega_{v}\partial M=\omega_{v}r\tau dA=\omega_{v}r^{2}\tau d\theta dr$$

(5) $$\partial Q=\overset{2\pi }{\underset{0}{\int }}\overset{R_{s}}{\underset{R_{p}}{\int }}\omega_{v}r^{2}\tau d\theta dr$$

where $R_{s}$ is the radius of the shoulder, $R_{p}$ is the radius of the pin, $\mu_{f}$ is the coefficient of friction, τ is the contact stress, $\omega_{v}$ is the angular velocity in $rad^{-1}$. Furthermore, we evaluated τ according to Coulomb’s law as follows:

(6) τ=μP

where P is the contact pressure. The plastic heat in a body is generated due to the plastic work performed on the shear layer during the sticking phase and can be defined as:

(7) $$H_{plas}=\eta \sigma {\dot{\varepsilon }}_{plas}$$

where $H_{plas}$ is the plastic heat generated, η is the thermal conversion efficiency, σ is the deviatoric stress, and ${\dot{\varepsilon }}_{plas}$ is the plastic strain rate. The heat is conducted from the stir zone (SZ) to the rest of the workpiece in the form of conduction, whereas the workpiece loses heat to the ambient surroundings in the form of convection and radiation heat. We considered a convection coefficient of $30\;W/{\mathrm{mm}}^{2}{}^{\circ}C$ on the workpiece surface. We considered radiation coefficients 0.09 and 0.12 for the AA6061 and AZ31B, respectively. The different thermal and mechanical boundary conditions are shown in Figure 1a. Frictional and plastic heat are generated where a tool contacts a workpiece. We modeled convective and radiative heat loss on all the workpiece surfaces except the bottom. The bottom surface was in contact with the backing plate; therefore, we modeled heat conduction between the bottom surface and the backing plate with a coefficient of $4000\;W/{\mathrm{mm}}^{2}{}^{\circ}C$ [21]. Different types of clamps can be used to contain the workpiece in the x and y directions, whereas the tool and backing plate hold the plate in position relative to the z axis (Figure 1a). The transient heat conduction equation is expressed as follows [22]:

(8) $$\nabla \cdot \left(k\left(T\right)\nabla T\right)=\rho C_{P}\frac{\partial T}{\partial t}$$

The convective and radiative heat losses are estimated as [22]:

(9) $$q=h_{conv}\left(T-T_{amb}\right)+\psi \sigma_{sb\;}\left(T^{4}-T^{4}{}_{at}\right)$$

where k is the thermal conductivity, $C_{p}$ is the specific heat capacity, $h_{conv}$ is the convective heat coefficient, ψ is the emissivity, $\sigma_{sb}$ is the Stefan–Boltzmann constant, T is the temperature variable, and $T_{at}$ is the ambient temperature. 

The coupled nature of the CEL demands that the conservative Lagrangian equations are converted to the Eulerian conservative equations, as broadly explained in the literature and represented as follows [23]:

(10) $$\frac{\partial \rho }{\partial t}+\nabla \cdot \left(\rho u\right)=0$$

(11) $$\frac{\partial (\rho u)}{\partial t}+\nabla \cdot \left(\rho u⊗u\right)=\nabla \cdot \sigma +\rho b$$

(12) $$\frac{\partial e}{\partial t}+\nabla \cdot \left(eu\right)=\sigma :D$$

where σ is the Cauchy stress, b is the body force, e is the total energy per unit volume, and D is the velocity strain. The Eulerian conservative Equations (8)–(10) can be represented in a general form as [21]:

(13) $$\frac{\partial \phi }{\partial t}+\Phi =S$$

where Φ is the flux function, and S is the source term. Using the operator splitting operation, Equation (11) is split as follows [24]:

(14) $$\frac{\partial \phi }{\partial t}=S$$

(15) $$\frac{\partial \phi }{\partial t}+\nabla \cdot \Phi =0$$

Equation (12) defines the Lagrangian step, whereas Equation (13) defines the Eulerian step. The conservation equations for the Eulerian and Lagrangian analysis are represented as:

(16) $$\frac{D\phi }{Dt}=\frac{\partial \phi }{\partial t}+u\cdot \left(\nabla \phi \right)$$

**Figure 2:**
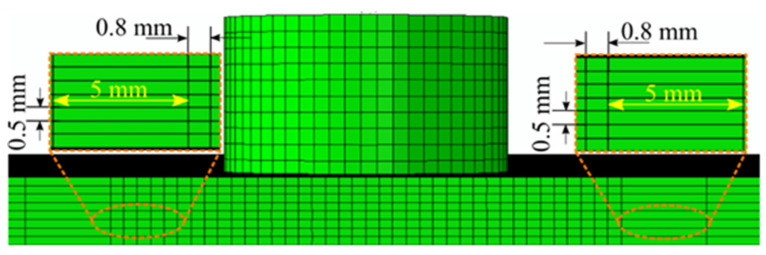
User-defined mesh of the computational domain.

Figure 2 shows the user-defined mesh of the computational domain. The Eulerian workpiece domain was meshed with three-dimensional, eight-noded EC3D8RT elements with reduced integration and thermal coupling. We meshed the SZ with a 0.8 mm element size in the traverse direction; further from the SZ, the mesh size was 5 mm [25]. We used a fine mesh size in the SZ to capture the flow of the material and dimensions of the surface and subsurface defects. We applied a coarse mesh size away from the SZ as we observed no defect formation at this location. The mesh sizes of various domains are shown in Figure 2. We performed the numerical analysis using an i7 processor with 16 GB RAM. The rotational and traverse velocities applied to the tool are summarized in Table 3. The thermophysical properties of AA6061 and AZ31B are summarized in Table 6 and Table 7, respectively. The properties beyond the temperature limits are extrapolated in simulation and are valid below the melting point temperature.

**Table 4:** The Johnson–Cook material model for AA6061 and AZ31B [26,27].

Materials	*A* (MPa)	*M* (MPa)	C	m	n	*T_room_* (°C)	*T_melt_* (°C)
AA6061	324	114	0.002	1.34	0.42	24	583
AZ31B	279	159	0.013	1.573	0.327	22	605

**Table 5:** The Johnson–Cook damage parameters for AA6061 and AZ31B [28,29].

Materials	d_1_	d_2_	d_3_	d_4_	d_5_
AA6061	0.071	1.248	1.142	0.147	0
AZ31B	−0.35	0.6025	−0.4537	0.206	7.2

**Table 6:** The thermo–mechanical properties of AA6061 [26].

T (°C)	25	100	149	201	260	316	371	427	482
E (GPa)	66.94	63.21	61.32	56.80	51.15	47.17	43.51	28.77	20.20
ν	0.33	0.334	0.335	0.336	0.338	0.36	0.40	0.41	0.42
$C_{p}\left({\mathrm{Jkg}}^{-1}{}^{\circ}C^{-1}\right)$	945	978	1000	1030	1052	1080	1100	1130	1276
$\rho \left({\mathrm{kgm}}^{-3}\right)$	2690	2690	2670	2660	2660	2630	263	2600	-
$\alpha \left({\mu \mathrm{mm}}^{-1}{}^{\circ}C^{-1}\right)$	23.5	246	25.7	26.6	27.6	28.5	29.6	30.7	-

**Table 7:** The thermo–mechanical properties of AZ31B [27].

T (°C)	20	75	100	125	150	200	250
E (GPa)	40.2	37.3	34.3	30.9	30.4	29.4	27.5
T (°C)	25	100	200	300	350	-	-
$k\left({\mathrm{Wm}}^{-1}K^{-1}\right)$	96.4	101	105	109	113	-	-
T (°C)	20	100	200	300	350	-	-
$C_{p}\left({\mathrm{Jkg}}^{-1}{}^{\circ}C^{-1}\right)$	1050	1130	1170	1210	1260	-	-
T (°C)	100	200	300	-	-	-	-
$\lambda \times 10^{-5}\left({}^{\circ}C^{-1}\right)$	2.64	2.70	2.79	-	-	-	-

## 4. Results and Discussion

The FSW tool inevitably deforms due to the plunging stage and continuous contact between the tool and the workpiece; because of its moderate dimensions, the tool pin deforms considerably. Tool wear is increased when performing dissimilar FSW compared with similar FSW [30]. Tool geometry primarily changes due to two factors: the various forces acting on the tool and the adhesion of the material from the workpiece to the tool body [31,32]. Although the tool wear is extensive with high-strength materials, substantial tool wear is also observed with lower-strength materials [33]. The wearing of the tool can lead to various mechanical and metallurgical defects. In the subsequent sections, we describe the changes in the tool profile with repeated usage at multiple process parameters and their effect on the quality of dissimilar welding. Furthermore, we employed a CEL model to investigate the various surface and subsurface defects and material mixing in dissimilar FSW.

### 4.1. Material Mixing

**Figure 3:**
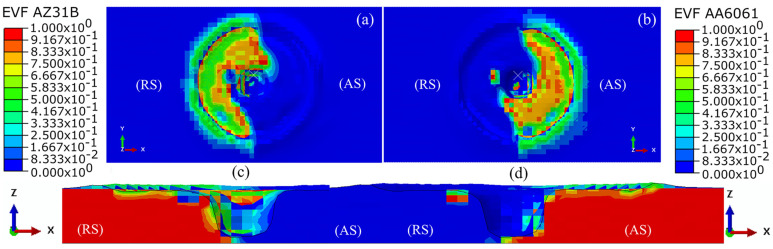
Numerical modeling of the material flow on top surface of SZ (**a**) RS (**b**) AS; and the cross-section (**c**) RS and (**d**) AS at end of the dwell stage at 900 rpm.

FSW produces a high-quality weld through various mechanical and metallurgical aspects, material mixing, and bonding. The material mixing of AA6061 and AZ31B is predicted in Figure 3 at the end of the dwell stage. We used the EVF approach to predict the presence of material on the RS (Figure 3a) and AS (Figure 3b) of the weld centerline. An EVF value of 0.5 indicates the presence of both materials of dissimilar welding in a single element. The material on the RS pushes into the AS with the rotation of the tool, while a similar proportion of the material from the AS is pushed into the RS. Figure 3c,d show the cross-sectional view of the material flow at the end of the dwell stage. Figure 3c indicates the presence of some AZ31B on the AS. In contrast, the presence of AA6061 on the RS is marginal in Figure 3d. This indicates the easy plasticization and better flow of material from the RS to the AS rather than from the AS to the RS at the end of the dwell stage. As the modulus of elasticity of AZ31B is lower than that of the AA6061, it was easily plasticized, rotated, and deposited around the dwelling tool.

**Figure 4:**
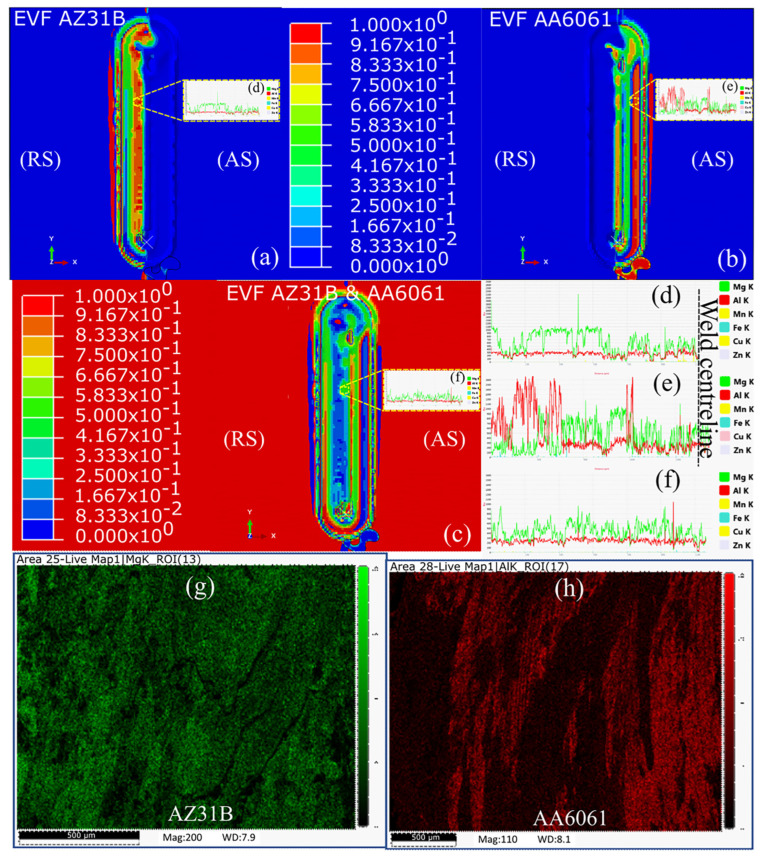
Numerical modeling of the material mixing on (**a**) RS, (**b**) AS, and (**c**) SZ; the EDS line scan on (**d**) RS, (**e**) AS, and (**f**) SZ; elemental mapping on (**g**) RS and (**h**) AS [Welding conditions 900 rpm and 30 mm/min].

Figure 4 shows the results of our numerical prediction and experimental investigation using an FESEM–EDS analysis of the material mixing and flow at the end of welding. We used an FESEM–EDS analysis to perform the line scan and area mapping. Figure 4a,b show a substantial amount of material mixing at the beginning of the weld length, i.e., at the end of the dwell stage. However, such material mixing was not evident for the remaining weld length. A higher concentration of the AZ31B was predicted near the weld centerline on the RS side. Simultaneously, a minor amount of AA6061 was predicted on the RS adjacent to the weld centerline. The small amount of AA6061 on the RS indicated the transfer of material from the AS with the rotation of the tool. Figure 4d shows the FESEM–EDS line scan on the RS of the welded sample. The higher AZ31B peaks indicated a higher presence of AZ31B on the RS. Moving toward the weld centerline, the elemental presence of the AZ31B decreased to be similar to that of AA6061, as predicted in Figure 4b. Furthermore, the presence of AA6061 was higher away from the weld centerline on the AS and reduced to be similar to that of AZ31B moving toward the weld centerline. At the center of the weld, the elemental compositions of AZ31B and AA6061 were almost similar, as also indicated by Figure 4f. Figure 4g,h show the FESEM–EDS area mapping on the RS and AS, respectively. The distribution of the material within the SZ was uniform. However, the presence of dark patches in Figure 4h indicates the intermittent presence of Mg within the SZ. The same was responsible for the sudden spike in the Mg presence in Figure 4e.

### 4.2. Defect Prediction

**Figure 5:**
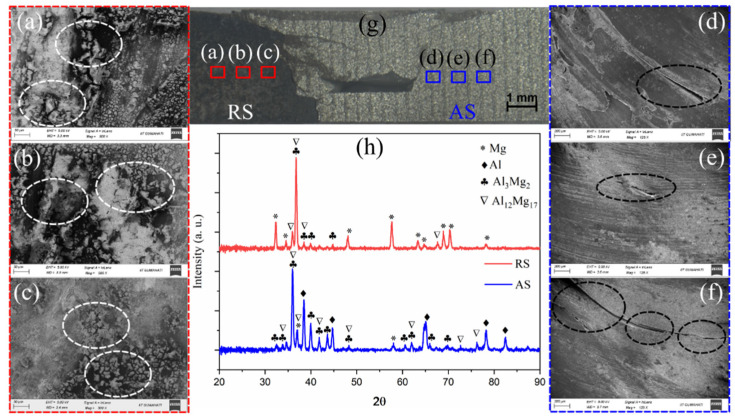
(**a**–**c**)—microcracks in SZ on RS; (**d**–**f**)—microcracks in SZ on AS; (**g**) a cross-section of AA6061-AZ31B SZ; (**h**) XRD patterns of RS and AS at 900 rpm and 30 mm/min.

Mechanical defects or metallurgical IMCs can weaken the joints, as shown in Figure 5. Figure 5 a–f show the microcracks on the RS and AS, respectively. The cracks on the AS were bigger than those on the RS. Figure 5g shows a cross-section of the weld in the SZ. The materials mixed, i.e., the dark-colored AZ31B on the RS mixed with the light-colored AA6061, on the AS. The mixing line between the two materials was visible. However, we observed several cracks on the RS and AS under an optical microscope (Figure 5g). We further observed the presence of these cracks under high-magnification FESEM. FSW samples are prone to IMCs, and high heat input increases the formation of IMCs. These IMCs can provide easy passages for the cracks to propagate [34,35]. Therefore, we analyzed the welded sample using X-Ray diffraction to observe the formation of IMCs (Figure 5h). The XRD pattern suggested the notable presence of ${\mathrm{Al}}_{3}{\mathrm{Mg}}_{2}$ and ${\mathrm{Al}}_{12}{\mathrm{Mg}}_{17}$ phases in the SZ of the welded sample. 

**Figure 6:**
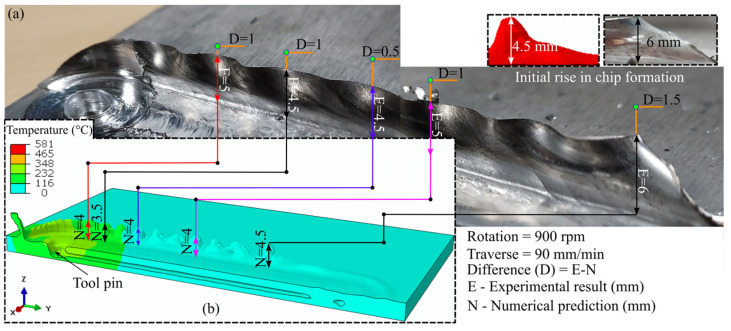
(**a**) The experimental investigation and (**b**) numerical modeling of flash formation on the retreating side at 900 rpm and 90 mm/min.

**Figure 7:**
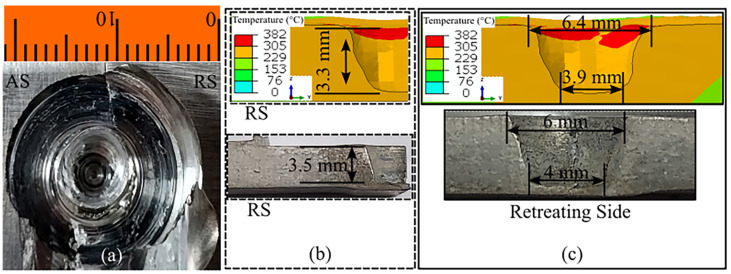
The experimental and numerical investigation of the exit hole: (**a**) experimental result, (**b**) height, and (**c**) cross-sectional variations in the solution domain.

The FSW process can produce various surface and subsurface defects in addition to the formation of the IMCs. Improper process parameters can lead to insufficient heat generation and improper material mixing, thus resulting in defective welding. Figure 6 shows the prediction of surface flash defects. Flash primarily forms due to the plunging action of the tool in the workpiece. Owing to the rotation of the tool, more material is deposited on the RS, resulting in increased flash formation (Figure 6a and Figure 7a). Thus, we considered the RS, i.e., the side consisting of AZ31B, in our analysis. We compared the predicted flash to the experimentally obtained value for the complete length of the welding. The maximum difference in pin height prediction was approximately 1.5 mm, observed at the beginning of the flash formation. The average difference in flash size prediction was approximately 1 mm compared with the experimental value. The difference was due to the mesh size consideration and human error during measurements. Figure 7 presents the unavoidable exit hole in FSW. The experimentally observed exit hole is shown in Figure 7a, whereas the height and cross-sectional width of the defect are presented in Figure 7b,c, respectively. We predicted the height of the defect with substantial accuracy. The difference between the experimental and numerical results was marginal, at approximately 0.2 mm. The exit hole diameters near the top and bottom surface of the workpiece showed minor deviations of approximately 0.4 and 0.1 mm, respectively. The refilling technique is used to repair the exit hole in similar FSW. However, the refilling technique can be a difficult approach due to the dissimilar nature of the weld zone in dissimilar FSW [36].

**Figure 8:**
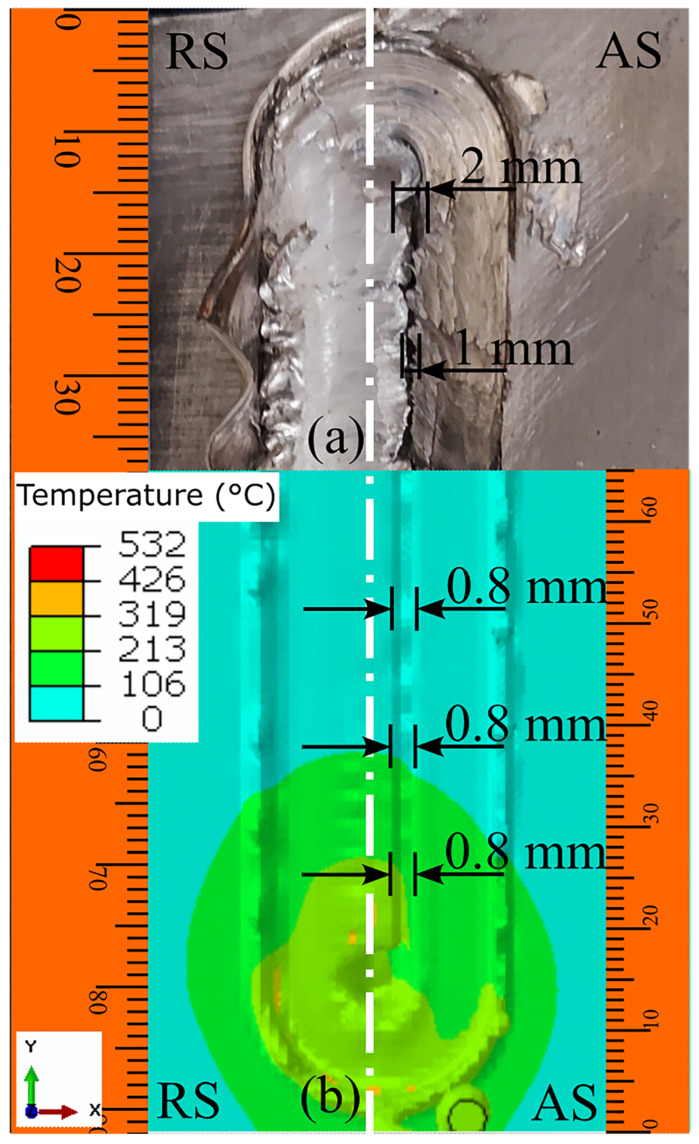
The (**a**) experimental and (**b**) numerical investigation of the surface tunnel generated at SZ during dissimilar AA6061–AZ31B FSW at 1200 rpm and 30 mm/min.

The tunnel defect is one of the most common defects in FSW. This defect generally occurs below the workpiece’s top surface. However, improper process parameter selection can also lead to tunnel defects on the surface. An example of a surface tunnel is depicted in Figure 8. Improper process parameters lead to insufficient heat generation and reduce material plasticization at the SZ. The maximum temperature under the tool shoulder and around the tool pin is approximately 400 °C. Thus, the maximum temperature is approximately 60% of the melting point temperature of the base materials; thus, this temperature is too low to properly plasticize the material. Moreover, the tool rotation and traverse are similar on the AS. Therefore, the tool, after leaving the surface tunnel defect behind, forcibly pushes out the material. Alternatively, as the material is deposited behind the tool with each rotation, this defect is not observed on the RS. We numerically predicted a continuous-length surface tunnel on the AS, and we experimentally observed a similar surface tunnel (Figure 8). The width of the experimental surface tunnel was approximately 1 mm throughout, whereas the numerically predicted result was approximately 0.8 mm. However, in a unique location at the beginning of the defect, the width of the defect was approximately 2 mm. The minor deviation between the predicted and experimental results may have occurred due to machine and measurement errors.

**Figure 9:**
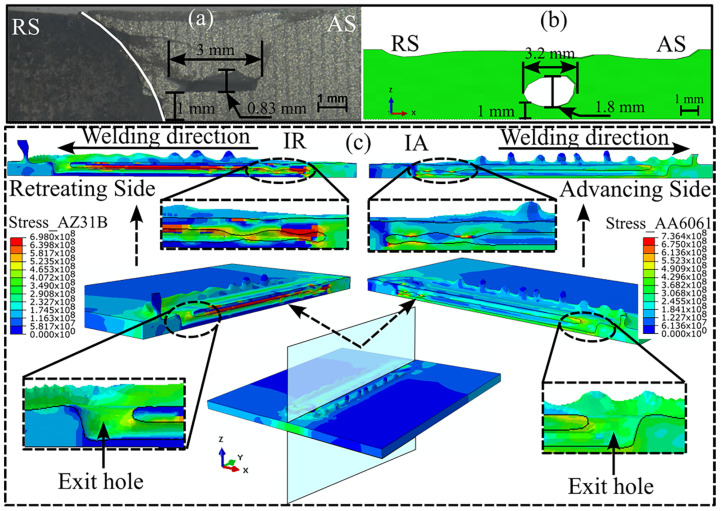
An illustration of the cross-sectional subsurface tunnel defect: (**a**) experimentally and (**b**) numerically modeled results; (**c**) the progress of the subsurface tunnel defect and stress distribution within the workpiece.

Figure 9 illustrates the subsurface tunnel defect appearing during FSW. Figure 9a shows the experimentally observed tunnel defect, whereas Figure 9b shows the numerically predicted result. The location and the width of the tunnel defect were accurately predicted. The height of the tunnel defect was numerically overpredicted. The possible reasons for this could be the mesh size considerations and the isotropic material properties that we used for the modeling. We split the workpiece longitudinally, as shown in Figure 9c, to observe the progress of the tunnel defect and stress distribution around the defect. The weld initiation site on the AS and RS, as denoted by IA and IR, respectively, showed a tunnel defect of varying depths; however, the defect depth became almost uniform thereafter. The generation of sufficient heat during the dwell stage could have been responsible for lesser defect depth at the weld initiation. We observed slightly higher stress on the AS than on the RS; however, the higher stress concentration on the AS localized to certain regions of the SZ. The stress was evenly distributed in the remainder of the plate on the AS. Alternatively, a higher stress concentrated near the top surface of the workpiece on the RS, whereas the stress near the bottom surface on the RS was almost negligible. This showed that most of the material was deposited around the top surface, and we observed much less material movement around the bottom surface. This is an important reason for the generation of tunnel defects. Furthermore, the use of a conical pin may have been a reason for the improper material movement near the bottom surface.

**Figure 10:**
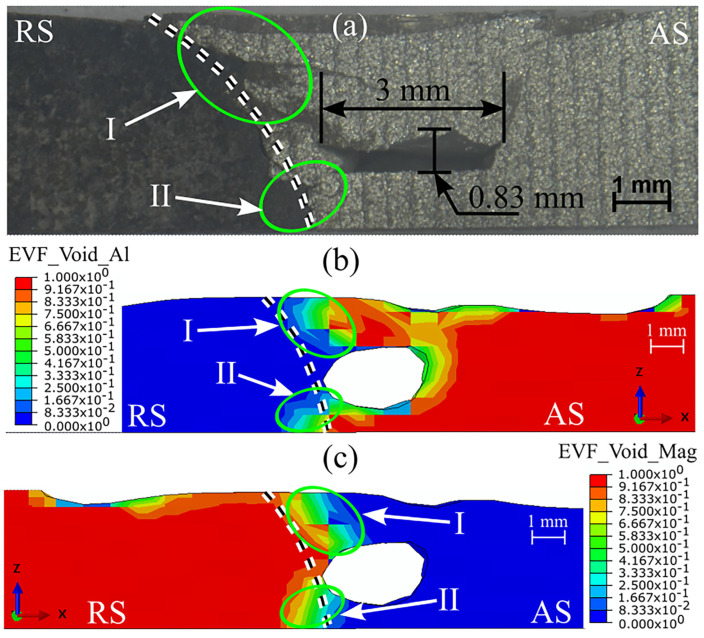
The visualization of the material mixing and prediction of the interface line in AA6061–AZ31B dissimilar FSW: (**a**) the experimental result, and (**b**) AA6061 and (**c**) AZ31B when welding at 900 rpm and 30 mm/min.

The material mixing pattern and interface line location are predicted in Figure 10. Figure 10a shows the experimental cross-section of the AA6061–AZ31B weld. The interface line between the materials is visible from the cross-section. Figure 10b,c predict the presence of AA6061 and AZ31B, respectively. The interface line between both materials was predicted on the RS. We observed material mixing primarily in the location denoted by I and II in Figure 10b,c. The tunnel defect was responsible for insufficient material mixing in the void region. Thus, both improper material mixing and the presence of defects lead to the weakening of the weld joint. The material mixing pattern in Figure 10 also indicates that more material flowed from the AS to the RS.

### 4.3. The Effect of Plate Position on the Material Flow

**Figure 11:**
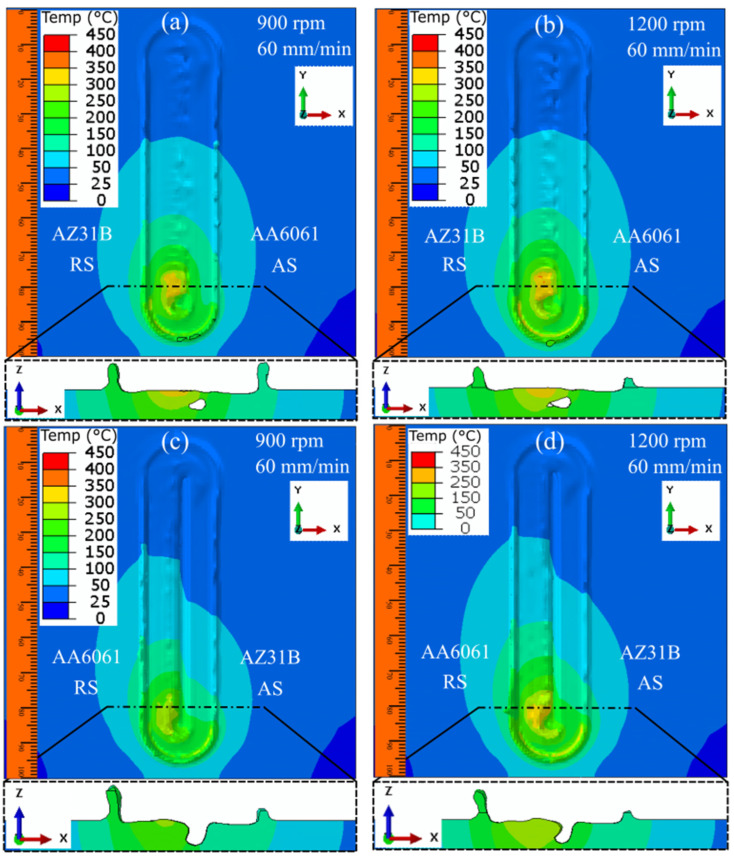
The numerical modeling of the nonuniform surface evolution during AA6061–AZ31B dissimilar FSW when the position of the base material was changed: (**a**) AA6061–AZ31B at 900 rpm and 60 mm/min, (**b**) AA6061–AZ31B at 1200 rpm and 60 mm/min, (**c**) AZ31B–AA6061 at 900 rpm and 60 mm/min, and (**d**) AZ31B–AA6061 at 1200 rpm and 60 mm/min.

Figure 11 presents the prediction of the surface defects during FSW for different process parameters when the position of the plates was interchanged between the AS and RS. The FSW with AA6061 on the AS produced a more uniform weld than welding with AZ31B on the AS. The higher heat generation and better tear-drop-shaped heat zone in Figure 11a,b were responsible for the better welding. However, the heat flow pattern was improper when we placed AZ31B on the AS, leading to the formation of a surface tunnel in both cases (Figure 11c,d). The results of the cross-sectional investigation near the weld completion for all four cases also indicated the presence of a surface tunnel with a depth of more than half of the workpiece thickness when we used AZ31B on the AS (Figure 11c,d). Alternatively, we observed no surface defects when we used AA6061 on the AS. Multiple researchers have recommended placing aluminum alloy on the AS and magnesium alloy on the RS [37,38,39]. The absence of a surface tunnel defect in Figure 11a,b indicates a better weld than welding with AZ31B on the AS, but the quality of the weld could be further improved. Multiple factors are responsible for defect generation in FSW. The change in base material position helped eliminate surface defects. Subsequently, proper heat generation and material flow can eliminate subsurface tunnel defects. These can be achieved by proper tool shape and size investigations. As the material flow improves with better tool design, subsurface tunnel defects will be eliminated, thereby improving the weld quality.

### 4.4. The Modification of Tool Profile Due to Wear

**Figure 12:**
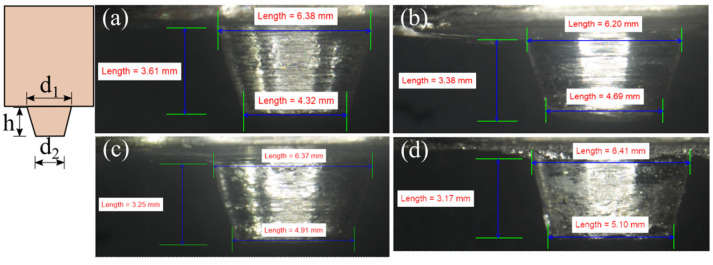
The progressive variation in the tool pin geometry when welding at 900 rpm and traverse speeds of (**a**) the initial tool under a no-weld condition, and under (**b**) 30, (**c**) 60, and (**d**) 90 mm/min.

**Figure 13:**
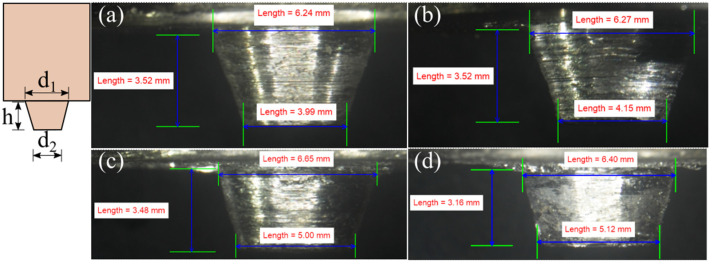
The progressive variation in the tool pin geometry when welding at 1200 rpm and traverse speeds of (**a**) the initial tool under a no-weld condition, and under (**b**) 30, (**c**) 60, and (**d**) 90 mm/min.

We performed FSW with AA6061 on the AS and AZ31B on the RS. The tool profile change after welding at 900 and 1200 rpm is presented in Figure 12 and Figure 13, respectively. We analyzed the tool profiles depicted in these figures after removing the adhered base material coating on the tool surface; i.e., the tool profile shown in Figure 12 and Figure 13 are actual tool profiles after welding. Figure 12a shows the initial tool condition, and Figure 12d shows the tool condition after welding completion at 900 rpm and 90 mm/min. The major pin diameter (d_1_), minor diameter (d_2_), and pin length (h) changed by 0.47%, 18.06%, and –12.19%, respectively, between the initial and final tool conditions (Figure 12). We noted a minor change in the volume of the pin, i.e., 82.114 and 82.773 mm^3^, between Figure 12a and Figure 12d. The slight difference in the volume may have occurred due to the small quantity of workpiece material on the tool and the measurement error during experimental measurements. The d_2_ dimension changed more due to its smaller diameter and initial and constant contact with the workpiece during the plunge stage. The same was responsible for reducing the height of the tool pin, which can lead to root defects due to improper tool pin penetration. The changes in the d_1_, d_2_, and h between Figure 13a and Figure 13d, i.e., the initial and the final tool conditions, were 2.56 %, 28.32%, and −10.23%, respectively. Furthermore, the volume of the tool pin (Figure 13d) was 82.639 mm^3^, which was a minimal difference of 0.134 mm^3^ from the tool pin shown in Figure 12d. The similar volume of the tool pin after equal numbers of weld runs, irrespective of the process parameters, indicated marginal material loss from the tool pin. In contrast, the tool pin shape drastically changed after each weld run (Figure 12 and Figure 13). Thus, we concluded that during dissimilar Al–Mg FSW, the primary concern for tool wear is the diminishing dimension of, rather than the material loss from, the tool surface. Table 8 summarizes the changes in the d_1_, d_2_, and h values after each weld run when welding at 900 and 1200 rpm.

**Figure 14:**
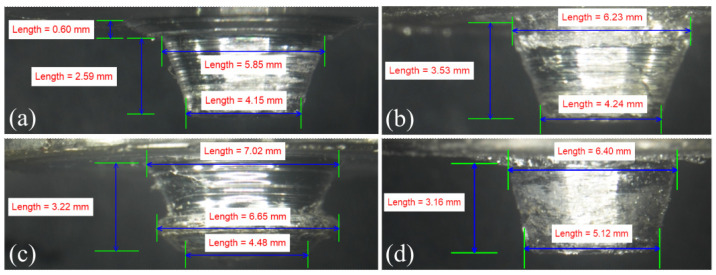
The variation in the tool pin geometry when welding at 1200 rpm: (**a**) the weld material adhered to tool surface; (**b**) the weld material was removed from the tool surface when the tool traverse speed was 60 mm/min; (**c**) the weld material adhered to the tool surface; and (**d**) the weld material was removed from the tool surface when the tool traverse speed was 90 mm/min.

**Figure 15:**
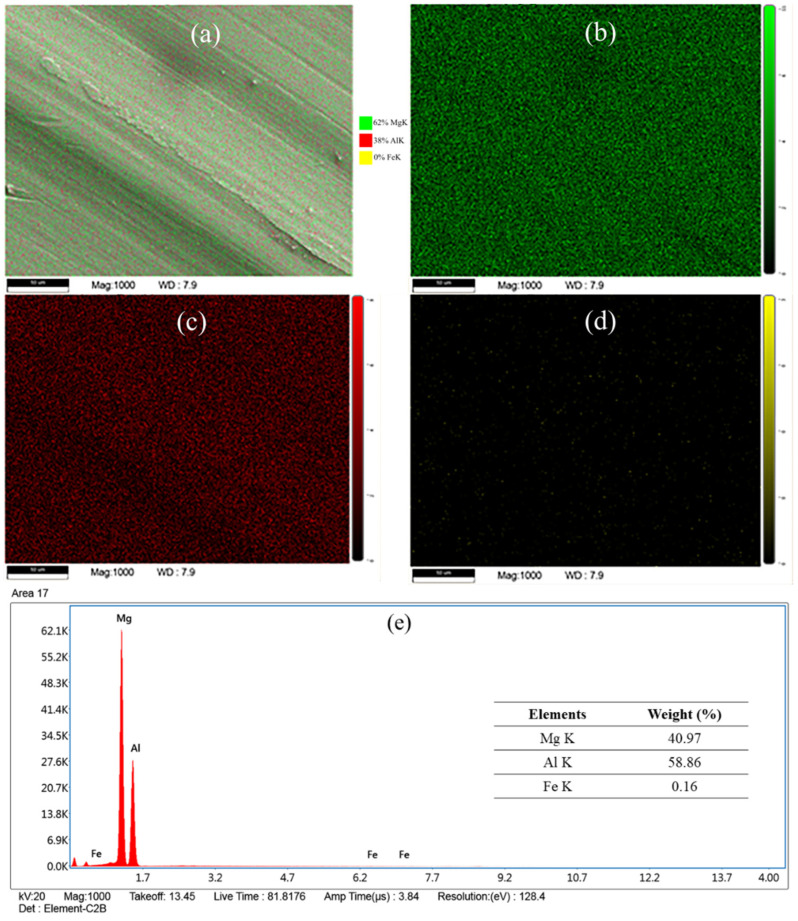
The scanning electron microscopy (EDS) mapping of (**a**) the workpiece material adhered to the tool during welding, showing the presence of (**b**) Mg, (**c**) Al, (**d**) Fe and (**e**) the intensity peak of the constituent materials.

Figure 14 shows the difference in the actual tool pin profile due to the adhesion of the workpiece material. Figure 14a,c show the material adhesion after welding at 60 and 90 mm/min, respectively; the tool rotation speed was 1200 rpm. The original pin shape after removal of the adhered material is shown in Figure 14b,d. The presence of foreign material on the tool was easily visible at the root of the tool pin (Figure 14a). The pin height decreased by approximately 0.6 mm due to the adhered material, while the d_1_ and d_2_ changed by −6.09% and −2.12%, respectively. Moreover, Figure 14c shows that the original conical shape of the tool pin changed into a mushroom shape due to the adhesion of the workpiece material [30]. This led to a substantial change in the original tool dimensions of d_1_ and d_2_, i.e., by 9.69% and −12.5%, respectively. We analyzed the material adhered to the tool pin using the FESEM–EDS analysis, as shown in Figure 15. Figure 15a indicates the uniform distribution of Mg, Al, and Fe, i.e., the main constituents of the AA6061, AZ31B, and tool steel, respectively, on the material adhered to the tool surface. Figure 15b–d show the distribution of the Mg, Al, and Fe on the surface of the adhered material. The results of the FESEM–EDS analysis indicated the uniform distribution of the Mg and Al on the top surface of the SZ, and a meagre presence of Fe was detected in the mixed material. Although we performed the FSW using a 0 mm tool offset, the tool surface contained a higher percentage of AA6061, i.e., 58.86%, than AZ31B, at 40.97% (Figure 15e). The results of the numerical modeling of the material mixing also indicated higher material movement from the AS to the RS (Figure 10). The Fe concentration was marginal (0.16%). This further indicated that the tool material was not eroded during welding.

#### The Tool-Wear-Induced Defects

**Figure 16:**
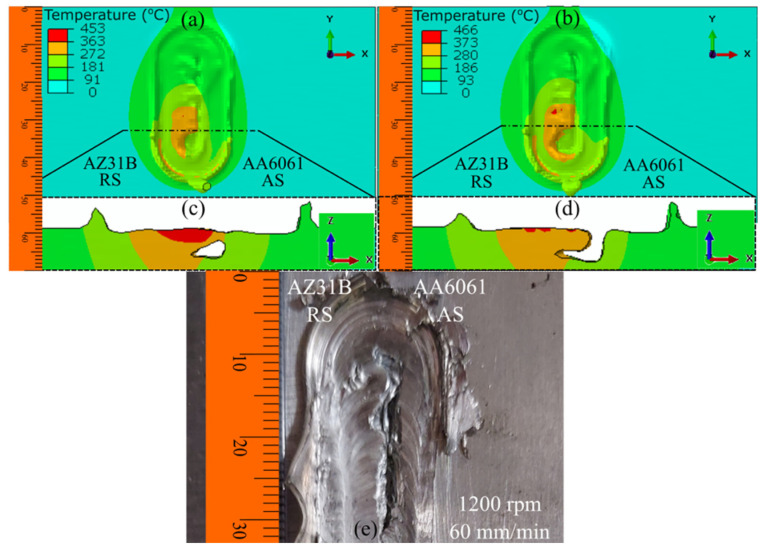
A comparative illustration of the progress of FSW at 1200 rpm and 60 mm/min with (**a**) the tool as shown in Figure 13a (without considering tool wear) and (**b**) the tool as shown in Figure 13c (with tool wear); (**c**) a cross-section view of the defect in Figure 16a and (**d**) a cross-sectional view of the defect in Figure 16b; and (**e**) the experiments on FSW.

Figure 16 shows the difference in the weld quality when modeling the initial 300 mm welding with the tool in different wear-out conditions, as described in Table 8. Figure 16a depicts the modeling with the initial unvarying tool condition, and the result predicts a subsurface tunnel defect, whereas surface tunnels are avoided. However, Figure 16b was modeled with the actual condition of the tool observed during experimentation (Figure 13c). The critical modeling with the actual condition of the tool generated a combination of defects, i.e., surface and subsurface tunnel defects (Figure 16b,d). We also observed a surface tunnel defect when welding at 1200 rpm and 60 mm/min using the moderately worn-out tool, as shown in Figure 16e. Therefore, we postulated that the quality and condition of the tool are crucial when numerically predicting weld quality. In Figure 16c, the sample is void of any surface defect due to the initial pin dimension, which allowed for a moderately easy flow of material around it. Alternatively, as the dimension of the tool modified due to tool wear after multiple uses, in Figure 16d, the surface tunnel defect also appeared because the tool was no longer capable of the sufficient plasticization or mixing of material around the tool pin.

## 5. Conclusions and Outlook

In this study, we focused on an experimental investigation of tool wear during dissimilar AA6061–AZ31B FSW. Moreover, we developed a coupled thermo–mechanical model and examined various surface and subsurface defects in dissimilar FSW. The experimental and numerical results were in close agreement. Furthermore, we numerically modeled the material mixing and validated the results with the help of the FESEM–EDS analysis. We also investigated the effect of plate position and different process parameters on the welding quality to derive the following major conclusions:

- The developed numerical model could predict the presence and variation of specific materials within the SZ. The same was validated by the FESEM–EDS results. The numerical model could successfully predict the material mixing and interface line on the surface and the welding cross-section;
- The model could simultaneously and accurately predict various surface and subsurface defects such as flash formation, exit hole, surface tunnel, and subsurface tunnel defects. The maximum deviations in flash height, exit hole diameter, surface tunnel width, and subsurface tunnel height prediction were 1, 0.4, 1.2, and 1 mm, respectively;
- The tool pin loses its dimensional integrity with multiple runs of welding. We observed major deviations in the tool height and minor diameter compared with the initial condition, whereas the change in the major diameter was negligible. The pin height changed by approximately 12.19% and 10.23% when welding at 900 and 1200 rpm, respectively. The minor diameter changed by 18.06% for 900 rpm and 28.32% for 1200 rpm;
- The tool shape also varies due to the material adhering to the tool from the SZ. The results of the FESEM–EDS analysis indicated the presence of more AA6061 (58.86%) from the AS compared with the AZ31B (40.97%) from the RS in the adhered material;
- We observed a surface tunnel with a height of more than 50% of the plate thickness when we used AZ31B on the AS and AAA6061 on the RS. The surface tunnel defect was absent when the we interchanged the position of the plate;
- The model could predict the difference in weld quality and defect formation when the condition of the tool was modified.
- In the course of this investigation, we focused on the development of a new model integrating the tool wear and dissimilar materials in the numerical modelling of FSW. More data on the different combinations of materials can illustrate the efficacy of the method, which deserves further investigation. In addition to the defects studied here, the surface topography and its comparison with the actual experiment is worth studying.

## Figures and Tables

**Table 1:** The chemical composition (wt%) of AA6061-T6 and AZ31B.

Material	Si	Cu	Fe	Zn	Mn	Al	Mg
AA6061	0.89	0.37	0.13	-	-	95.59	2.21
AZ31B	-	-	-	0.71	0.15	3.07	94.76

**Table 2:** The chemical composition (wt%) of H13 tool steel.

Element	Si	Mo	V	Cr	Mn	Co	W	Fe
(wt%)	1.89	1.81	0.60	4.72	0.62	1.03	1.98	87.35

**Table 3:** Details of the experimental process parameters and plate positions.

Weld No.	Plate Positioning (AS-RS)	RPM	Traverse Speed (mm/min)
Weld-I1	Al-Mg	900	30
Weld-I2	Al-Mg	900	60
Weld-I3	Al-Mg	900	90
Weld-II1	Al-Mg	1200	30
Weld-II2	Al-Mg	1200	60
Weld-II3	Al-Mg	1200	90

**Table 8:** The alterations in pin length, and the major and minor diameters of the conical pin with successive weld runs.

Weld No.	Pin Length (h) (mm)	Pin Length Change (%)	Pin Major Diameter (d_1_) (mm)	Pin Major Diameter (d_1_) Change (%)	Pin Minor Diameter (d_2_) (mm)	Pin Minor Diameter (d_2_) Change (%)
Tool-I	3.61	-	6.38	-	4.32	-
Weld-I1	3.38	−6.37	6.20	−2.82	4.69	8.56
Weld-I2	3.25	−9.97	6.37	−0.16	4.91	13.66
Weld-I3	3.17	−12.19	6.41	0.47	5.10	18.06
Tool-II	3.52	-	6.24	-	3.99	-
Weld-II1	3.52	0.00	6.27	0.48	4.15	4.01
Weld-II2	3.48	−1.14	6.65	6.57	5.00	25.31
Weld-II3	3.16	−10.23	6.40	2.56	5.12	28.32
